# State Causality and Adaptive Covariance Decomposition Based Time Series Forecasting

**DOI:** 10.3390/s23020809

**Published:** 2023-01-10

**Authors:** Jince Wang, Zibo He, Tianyu Geng, Feihu Huang, Pu Gong, Peiyu Yi, Jian Peng

**Affiliations:** 1 College of Computer Science, Sichuan University, Chengdu 610065, China; 2 College of Innovation and Entrepreneurship, Shijiazhuang Institute of Technology, Shijiazhuang 050228, China

**Keywords:** state causality, adaptive covariance, Cholesky decomposition, time series long-term forecasting

## Abstract

Time series forecasting is a very vital research topic. The scale of time series in numerous industries has risen considerably in recent years as a result of the advancement of information technology. However, the existing algorithms pay little attention to generating large-scale time series. This article designs a state causality and adaptive covariance decomposition-based time series forecasting method (SCACD). As an observation sequence, the majority of time series is generated under the influence of hidden states. First, SCACD builds neural networks to adaptively estimate the mean and covariance matrix of latent variables; Then, SCACD employs causal convolution to forecast the distribution of future latent variables; Lastly, to avoid loss of information, SCACD applies a sampling approach based on Cholesky decomposition to generate latent variables and observation sequences. Compared to existing outstanding time series prediction models on six real datasets, the model can achieve long-term forecasting while also being lighter, and the forecasting accuracy is improved in the great majority of the prediction tasks.

## 1. Introduction

Time series forecasting has long been a research focus. With the development of information technology, a significant amount of time series is generated in various production activities. As the output of the observation system, the time series, which is characterized by a large scale, objectively records the system’s information at each point in time, such as exchange rates and energy load. It has become a hot research topic to accurately mine the generation pattern and achieve high-precision long-term forecasting in large-scale time series. Traditional time series forecasting methods are based on statistical knowledge. Box and Jenkins [1] illustrated that the ARIMA model is theoretically applicable to time series analysis in various production activities.

In recent years, RNNs [2,3], which are essentially nonlinear exponential smoothing [4], have achieved satisfying results in sequence prediction for their ability to fit nonlinear relationships in short-term series. However, cumulative errors appear as the major drawback in long-term forecasting for large-scale time series in the models discussed above. Transformers [5,6] have fixed the problem of cumulative errors to some extent by optimizing the attention calculation method and decomposition-based strategy. However, it is firmly dependent on the periodicity of the series. In summary, the correlation between local data is crucial to the prediction results.

It shows that non-stationary properties are evident [7] from a survey of large-scale time series on exchange rates, diseases, and electrical loads. Moreover, similar to HMM [8], the observation sequence is dominated by hidden states. For example, temperature changes are influenced by weather and seasonal factors. VSMHN [9] and DGM2 [10] identify the hidden states by VAE [11,12], which applies neural networks to encode the latent variables [13] but is only suitable for the single-step generation of sparse sequences. It can be concluded that achieving long-term prediction for large-scale time series has the following challenges:

- Challenge 1: Large-scale time series have evident non-stationary properties, and nonlinear models, such as neural networks, rely heavily on data periodicity. As a result, it is a challenge to investigate the typical generation rule of large-scale series and improve the model’s generalization;
- Challenge 2: Hidden states influence the observation value of each moment, and the time span of a large-scale time series is larger. Therefore, it is a key issue to extend the hidden state estimation of time series from a certain moment to a period of time;
- Challenge 3: Time series depend on the values observed before. Another challenging assignment for the long-term forecasting of large-scale time series is how to retain temporal dependence in order to address the cumulative error problem.

To meet the above challenges, this paper proposes the State Causality and Adaptive Covariance Decomposition-based Time Series Forecasting (SCACD) model for long-term forecasting of the large-scale time series. SCACD uses latent variables to designate hidden states, builds the neural network for estimating the distribution of latent and observed variables in large-scale scenarios drawing on the idea of VAE, and finally generates prediction sequences. The main works are as follows:

1. For Challenges 1 and 2, this paper first extracts sub-sequences by sliding window, secondly extends time points to larger scale sequences, then finally, designs an adaptive estimation method to encode the latent variables corresponding sub-sequences, and finally uses causal convolutional networks to predict future latent variables.
2. For Challenge 3, SCACD employs the Cholesky [14,15] decomposition of the covariance matrix to maintain the temporal dependence, thus generating the latent variables. Based on the latent variables, SCACD infers the prior distribution of the observed variables and generates an observation sequence with the same approach.
3. SCACD’s effectiveness was validated and analyzed on six publicly available standard large-scale datasets.

## 2. Related Work

With the development of deep learning techniques, time series forecasting algorithms are also constantly improving. There are three main research subtopics of time series forecasting tasks, from short-term forecasting of early sparse time series to long-term forecasting of large-scale time series: autoregressive models, Transformer-based models, and causal convolution-based models.

### 2.1. Autoregressive Model

Shallow autoregression: This category contains ARIMA, exponential smoothing [4], and Kalman filtering [2,16,17], as well as Hidden Markov Model-based models (HMM) [8] for identifying hidden states. Box-Jenkins [1] illustrates that ARIMA could achieve high prediction accuracy by estimating the model’s unknown parameters after converting the series into a stationary series. The principle of exponential smoothing is to decompose a time series into numerous components, model them separately, and then combine them to obtain the forecast series. Kalman filtering considers the deviation between the true values and observation values, utilizing multidimensional known information to infer more essential data. To forecast future hidden states and time series, the HMM [18,19] estimates the state transition probability matrix and observation matrix.

Deep autoregression: Deep models, such as RNN [20], have achieved better results compared to shallow models in sequence tasks. Although RNNs can fit nonlinear short-term series, they could not be more effective in the long-term prediction of large-scale time series. In addition, deep autoregressive models also include generation-based models such as DGM, VSMHN, DeepAR [21], and others, among which DeepAR is the most representative, which predicts the value of each moment by predicting the corresponding distribution. Therefore, we can conclude that the correlation between the time series plays a key role in the time-series prediction. However, the above models do not address the cumulative error of long-term forecasting for large-scale time series. Consequently, although the autoregressive model is applicable to most forecasting scenarios, long-term forecasting of large-scale time series still has a low prediction accuracy.

### 2.2. Transformer-Based Model

Transformer [6] has been utilized successfully in the field of sequences. Differing from autoregressive models, Transformer adds attention and positional encoding to achieve parallel computing. Recent research has revealed that the Transformer-based models perform exceptionally well in long-term time series prediction.

Thus far, the enhanced Transformer-based models for long-term forecasting include FEDformer [5], Autoformer [22], Informer [23], Reformer [24], LST [25], LogTrans [26], etc. This kind of algorithm primarily improves the attention calculation method, thus increasing the accuracy and reducing the time complexity. In addition, the position encoding is replaced by the timestamp encoding in the interim, increasing forecast accuracy. FEDformer calculates the attention based on the frequency components of time series by Fourier analysis, which significantly improves the results of long-term series prediction, but it is computationally expensive. Autoformer considers series autocorrelation to calculate the attention score in the Transformer module based on the decomposition strategy. Informer employs a distilled self-attention mechanism and multi-scale timestamp encoding. Reformer increases the time complexity through locality-sensitive hashing attention and space complexity through RevNet. The logTrans adds convolutional self-attention layers, reducing memory from $O(L^{2})$ to $O(L{\left(\log L\right)}^{2})$.

These models show that sequence dependencies can be modeled for long-term forecasting [27], but rely heavily on periodicity. In general, our findings suggest that the correlation between subsequences is crucial.

### 2.3. Causal Convolution-Based Model

General neural network prediction algorithms for time series, such as LSTM, DRU, RNN, etc., model historical data and then implement multi-step prediction, resulting in significant cumulative error. Causal convolution [28,29] and dilated convolution [30] solve this problem to some extent, which captures the autocorrelation of the time series. The dilation mechanism [31] is introduced to expand the field of perception of the causal network by adjusting the distance between the convolution kernel elements or increasing the dilation rate. The primary sequence methods based on causal convolution are Wavenet [29] and TCN [32]. Unlike RNNs, TCNs are convolutional neural networks that serve as convolutional prediction models for time series. TCNs overcome the cumulative error problem for long-term time series prediction to some extent by combining causal and dilated convolution. The essential reason is to implement causal convolutions at multiple scale sequences from bottom to top, thus extracting autocorrelation across multiple scale sequences and expanding the field of perception. TCN models the lowest scale data, therefore, its prediction scale is limited to the number of dilation convolution layers, and as the scale of the time series increases, the applicability decreases. In conclusion, while causal convolution can capture sequence dependency with fewer parameters, existing models continue to struggle with a long-term prediction of large-scale time series.

## 3. Materials and Methods

Problem Definition: Given a time series $X=(x_{1},\ldots ,x_{i},\ldots ,x_{L})$, $x_{i}\in R$, the goal is to learn a mapping that predicts $\hat{X}=\left(x_{L+1},\ldots ,x_{L+n}\right)$, i.e., $\hat{X}=f\left(X\right)$. Since *X* is generated by some random process involving unobserved continuous random latent variable *a*. The process consists of two steps [11]: (1) $a_{i}$ is generated from a prior distribution $p(a_{i})$; (2) $x_{i}$ is generated from a conditional distribution $p(x_{i}|a_{i})$. To complete the forecasting based on these two steps, we propose an end-to-end SCACD composed of neural network components for large-scale time series. For long-term forecasting, a sliding window [1], which has two hyper-parameters: the size of the window (*l*) and the sliding step (step), is employed to extract the subsequences $S=(s_{1},\ldots ,s_{i},\ldots ,s_{T})$, $s_{i}\in R^{l}$. $s_{i}$ corresponds to the latent variable $z_{i}\in R^{h}$, where *h* is the dimension of $z_{i}$, i.e., *S* corresponds to $Z=(z_{1},\ldots ,z_{i},\ldots ,z_{T})$.

**Figure 1:**
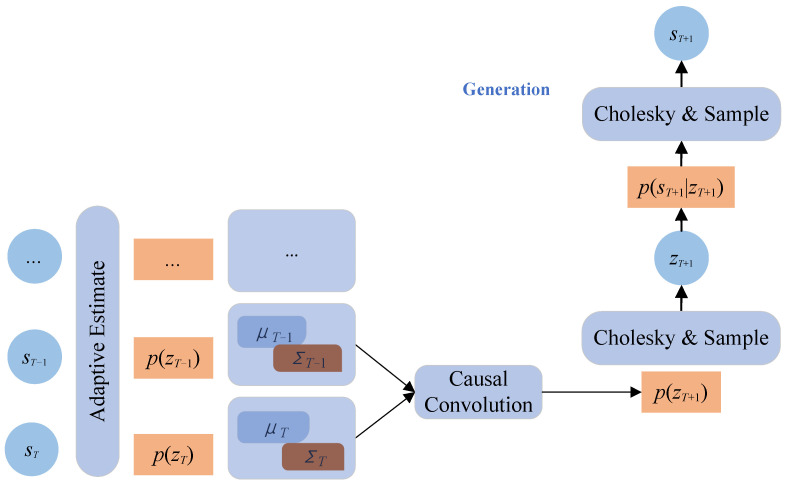
The schema of SCACD. It mainly includes three parts: Adaptive Estimation, State Causal Convolution, and Cholesky Decomposition.

First, SCACD adaptively estimates the posterior distribution $p(z_{i}|s_{i})$ of the latent variable $z_{i}$, assuming $z_{i}∼N\left(\mu_{z,i},\Sigma_{z,i}\right)$, and the $\left(\mu_{i},\Sigma_{z,i}\right)$ serves as the feature encoding of $z_{i}$. Furthermore, SCACD employs causal convolution to predict the $p(z_{T+1})$ of the future latent variable $z_{T+1}$ and generates $z_{T+1}$ after executing the Cholesky decomposition of the covariance matrix $\Sigma_{z,T+1}$. Finally, SCACD again adaptively estimates the conditional distribution $p(s_{T+1}|z_{T+1})$ for the future sequence $s_{T+1}$, $s_{T+1}∼N\left(\mu_{s,T+1},\Sigma_{s,T+1}\right)$. The $s_{T+1}$, similar to $z_{T+1}$, is generated by sampling after Cholesky decomposition. The diagram of the SCACD model is shown in Figure 1.

### 3.1. Adaptive Estimation for $p(z_{i}|s_{i})$

Based on the observed sequence $s_{i}$, we infer the posterior distribution $p_{\vartheta }\left(z_{i}|s_{i}\right)$ of the latent variable, whose distribution parameters are $\mu_{z,i}$ and $\Sigma_{z,i}$. However, the existing predecessors [9,10] assume that the dimensions of the latent variable are independent of each other. Although this hypothesis saves computational effort, it fails to account for the correlation between dimensions, resulting in information loss. For this problem, SCACD designs an adaptive algorithm for estimating the mean and covariance matrix. The processes are shown in the formulas:

(1) $$H_{z,i}=MLP\left(s_{i}\right)$$

(2) $$\mu_{z,i}=MLP\left(s_{i}\right)$$

(3) $$\Sigma_{z,i}=W\left(H_{z,i}-\mu_{z,i}\right)\cdot {\left(W\left(H_{z,i}-\mu_{z,i}\right)\right)}^{T}$$

**Figure 2:**
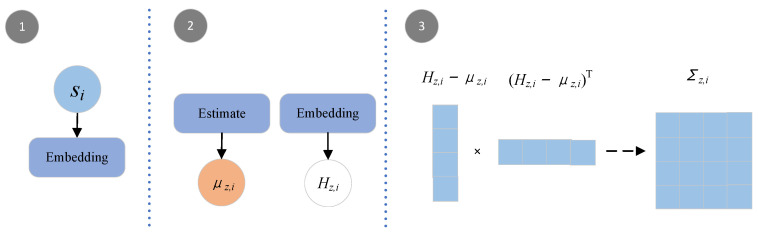
The phase of adaptive estimation. (1) Find the embedding of $s_{i}$; (2) Estimate $\mu_{z,i}$ and $H_{i}$; (3) Get $\Sigma_{z,i}$.

Through the adaptive estimation module, the encoding sequences of *Z* are obtained: $E_{\mu }=\left(\mu_{z,1},\ldots ,\mu_{z,i},\ldots ,\mu_{z,T}\right)$, $\mu_{z,i}\in R^{h}$, $E_{\Sigma }=\left(\Sigma_{z,1},\ldots ,\Sigma_{z,i},\ldots ,\Sigma_{z,T}\right)$, $\Sigma_{z,i}\in R^{h\times h}$. The schematic of the adaptive estimation is shown in Figure 2.

### 3.2. State-Causal Convolution

**Figure 3:**
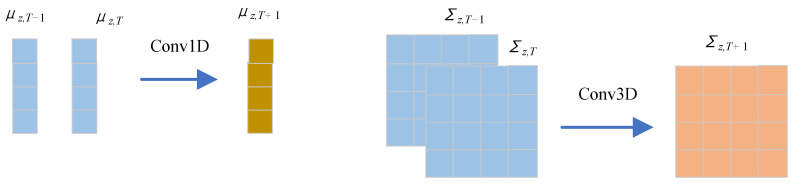
State causal convolution. The mean and covariance matrix of $z_{T+1}$ are estimated using 1D and 3D convolutions, respectively.

Following the idea of second-order Markov chains [33], we use the encoding of the first two states to forecast the next state, i.e., the *T* is set to 2. Specifically, utilizing causal convolutions, the encodings of $z_{T-1}$ and $z_{T}$ are used as input to estimate the mean and covariance matrix of $z_{T+1}$: $\mu_{z,T+1}$ and $\Sigma_{z,T+1}$. The convolution processes are in the following formulas, and the diagram is shown in Figure 3.

(4) $$\mu_{z,T+1}=Conv1D\left(E_{\mu }\right)$$

(5) $$\Sigma_{z,T+1}=Conv3D\left(E_{\Sigma }\right)$$

### 3.3. Sequence Generation

#### 3.3.1. Decomposition and Sampling

**Figure 4:**
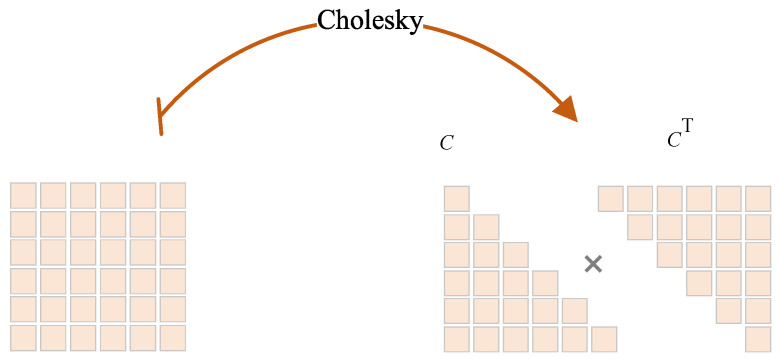
Cholesky decomposition. The covariance matrix is decomposed to the diagonal matrix, followed by sampling $z_{T+1}$.

SCACD infers the prior distribution $p_{\pi }\left(s_{T+1}|z_{T+1}\right)$ to generate future sequences, where $z_{T+1}$ is generated as $p_{\varphi }\left(z_{T+1}\right)$. Hence $z_{T+1}$ is crucial in putting the forecast into practice. Since $z_{T+1}$ is assumed to follow the Gaussian distribution: $z_{T+1}∼N\left(\mu_{z,T+1},\Sigma_{z,T+1}\right)$, SCACD generates $z_{T+1}$ via Cholesky decomposition [14,15], resulting in efficient sampling. Positive semidefiniteness is necessary for Cholesky decomposition, $\Sigma_{z,T+1}$ is multiplied by $\Sigma_{z,T+1}^{T}$. The specific procedures are shown in Equation (6) and Figure 4.

(6) $$\begin{array}{cc} z_{T+1} & =\xi \cdot C+\mu_{z,T+1}\\ {\hat{\Sigma }}_{z,T+1} & \overset{Cholesky}{=}CC^{T}\\ {\hat{\Sigma }}_{z,T+1} & =\Sigma_{z,T+1}\cdot \Sigma_{z,T+1}^{T} \end{array}$$

where *C* belongs to the lower triangular matrix, ξ∼N(0,I).

#### 3.3.2. Sequence Prediction

Based on $z_{T+1}$, SCACD utilizes MLP [11] to estimate the prior conditional distribution $p_{\pi }\left(s_{T+1}|z_{T+1}\right)$, as follows:

(7) $$\begin{array}{cc} \Sigma_{s,T+1} & =W\left(H_{s,T+1}-\mu_{s,T+1}\right)\cdot {\left(W\left(H_{s,T+1}-\mu_{s,T+1}\right)\right)}^{T}\\ \mu_{s,T+1} & =MLP(H_{s,T+1})\\ H_{s,T+1} & =MLP(z_{T+1}) \end{array}$$

Finally, the forecasting sequence ${\hat{s}}_{T+1}$ is then obtained by re-implementing Cholesky-based sampling and adding an attention layer. The final objective function is as follows:

(8) $$L\left(\vartheta ,\varphi ,\pi \right)=\frac{1}{N}\sum_{n=1}^{N}(∥{\hat{s}}_{T+1,n}-s_{T+1,n}{∥}_{2}^{2})$$

(9) $${\hat{s}}_{T+1}=\frac{1}{K}\sum_{k=1}^{K}s_{T+1}^{k}$$

where *n* denotes the sample serial number, and $s_{T+1}^{k}$ is sampled *K* times. ϑ, φ, and π are the parameters to be optimized.

## 4. Results and Discussion

We conducted extensive experiments to evaluate the effectiveness of SCACD: comparison of prediction performance, covariance matrix analysis, analysis for the dimension of *z*, and analysis of efficiency. Moreover, six real datasets were calculated to certify the generality of SCACD.

### 4.1. Hyperparameter for SCACD

Pytorch 1.5, NVIDIA GeForce GTX 1060, and the Core i7-7700HQ CPU are the environments for implementation. The *h* is set as 16, the hidden layers for MLP are set as 3, the number of convolution layers is 1, and the *K* ranges from 5 to 50. SCACD was optimized by Adam optimizer [34], with the learning rate decaying by 50% every 100 epochs to avoid the local optimum. The parameters are suitable for most prediction tasks.

### 4.2. Introduction of the Models

- FEDformer [5], being a Transformer-based model, computes attention coefficients in the frequency domain in order to represent point-wise interactions. Currently, FEDformer is the best model for long-term prediction, regardless of processing efficiency.
- ARIMA [35] is a linear model that includes fewer endogenous variables and is mainly used for short-term forecasting of stationary time series.
- LSTM [36] is a deep model for sequence prediction, which can capture the nonlinear relationship and solve the problem of RNN gradient disappearance by the memory unit.
- TCN [28] utilizes causal and dilated convolution to capture the multi-scale temporal features of time series, which can build deep networks. The method is effective in time series multi-step prediction.
- DeepAR [21] does not directly output definite prediction values but estimates the probability distribution of the future values to predict the observation sequence.
- GM11 [37] is obtained by constructing and solving the differential equation of the cumulative series, which has the advantage of fewer prior constraints on the series.

### 4.3. Introduction to Datasets

The datasets for validating the algorithms include Electricity Transformer Temperature (ETT) [38], Exchange [25], Electricity, Weather, Traffic and Influenza-Like Illness (ILI) [22]. The details of the datasets are shown in Table 1, we divide the six datasets into training: test: val in a 7:2:1 ratio.

### 4.4. Comparison of Prediction Performance

Recently, Transformer-based models, such as FEDformor, Autoformer, and Informer, have performed best in long-term forecasting tasks. In order to verify the effectiveness of SCACD and make a fair comparison, the experiment set up the same six prediction tasks from the FEDformer to evaluate the efficiency. Two historical sliding windows are used to predict the future window sequence. Therefore, the sliding step size is the actual prediction length. The (*l*, step) corresponding tasks are, respectively, (800, 720), (350, 336), (200, 192), and (100, 96) for the datasets except for ILI, which is set as (62, 60), (50, 48), (38, 36), and (26, 24). The results are shown in Table 2. In general, SCACD has the best performance in most of the prediction tasks.

**Retrospective Analysis.** The FEDformer performed better in ETT-96, ETT-192, ETT-336, Electric-192, and Electricity-336. The investigation discovered that ETT and Electricity are more cyclical and that FEDformer reinforces the embedding with the timestamp, which improved prediction accuracy.

ARIMA is effective for short-term stationary time-series prediction, but it cannot handle the accumulative error problem of long-term non-stationary time-series prediction. TCN designs causal convolution and extended causal convolution to extract multi-scale features for a short-term forecast, but the generational rule of the time series is not mined. DeepAR mainly focuses on single-point prediction, ignoring the influence of the hidden state on the observation series, resulting in a poor long-term prediction effect. The GM is obtained by solving the differential equation of the cumulative series, which has fewer prior constraints on the series. However, it is less effective for long-term prediction.

**Ablation Analysis.** The SCACD-NC (SCACD without Cholesky decomposition) in Table 2 is the strategy assuming that the dimensions of the latent variable are independent. The results show that SCACD is superior to SCACD-NC except for Exchange-336, Weather-336, and ILI-48. The energy loss caused by the assumption of independence limits SCACD-NC’s generating ability. Furthermore, when compared to other models, SCACD-NC shows significant advantages. It exhibits the rationale and robustness of the state causality-based sequence generation algorithm for long-time series prediction tasks.

**Figure A1:**
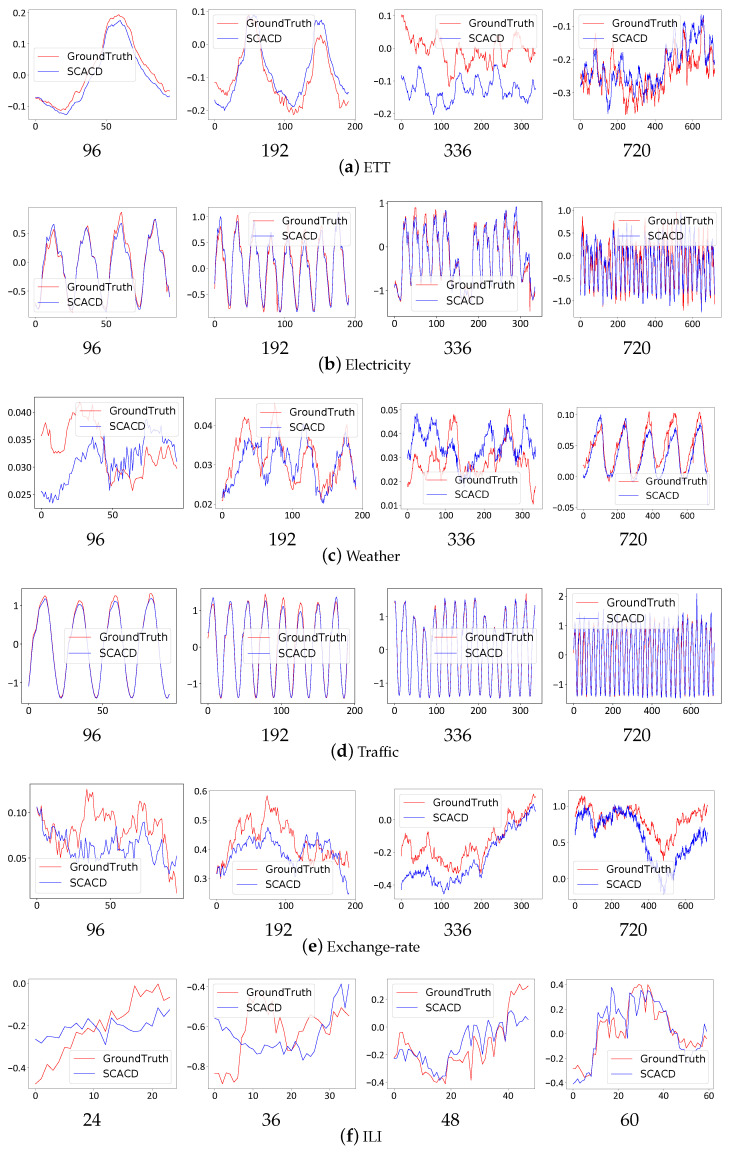
The forecasting curves of the six datasets.

The advantages of SCACD are: (1) The covariance matrix is adaptively estimated to capture the correlation between the dimensions of the variable, which improves the generation accuracy of variables; (2) SCACD predicts the distribution of the future variables with fewer parameters by state causal convolution. In addition, in datasets with weak periodicity, SCACD’s prediction effect outperforms other methods. The visualizations of the predictions are presented in Figure A1.

### 4.5. Covariance Matrix Analysis

**Figure 5:**
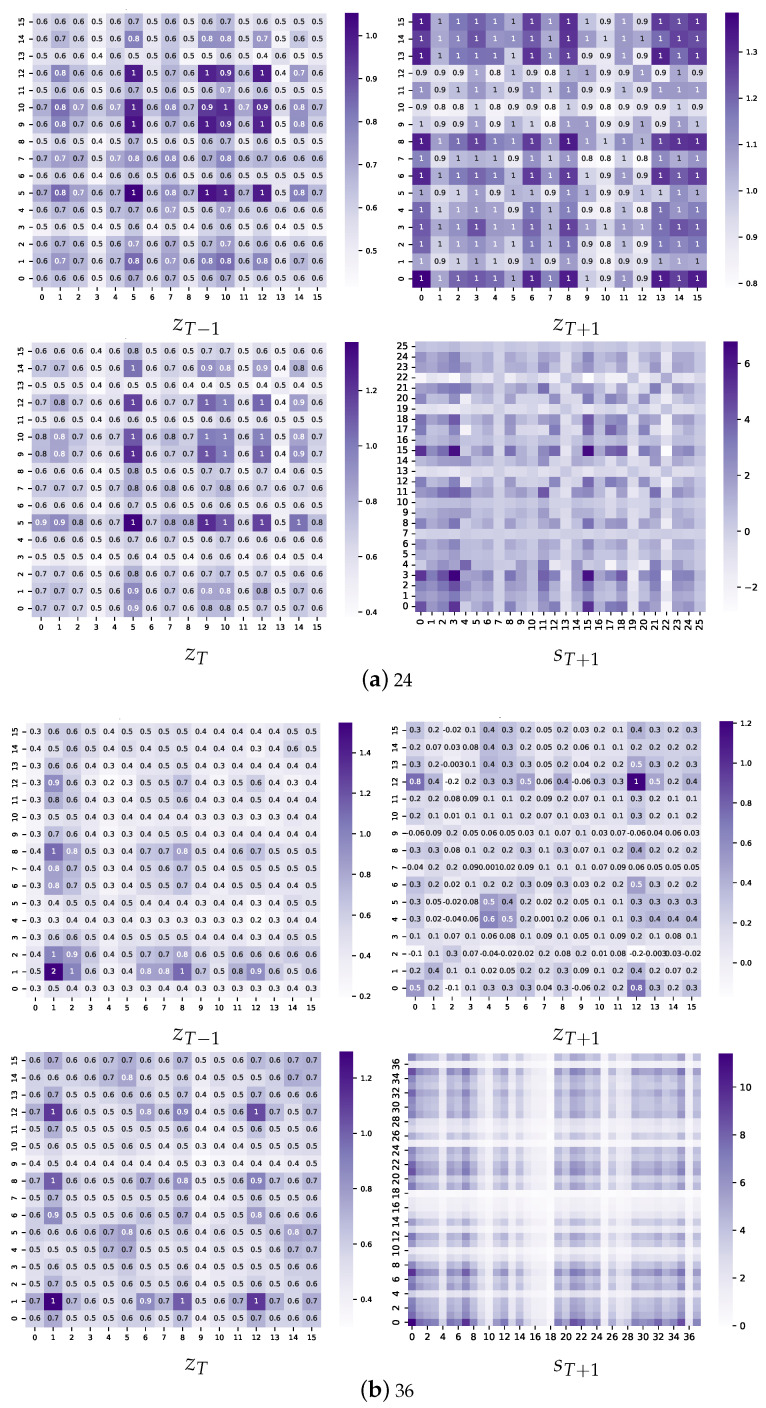

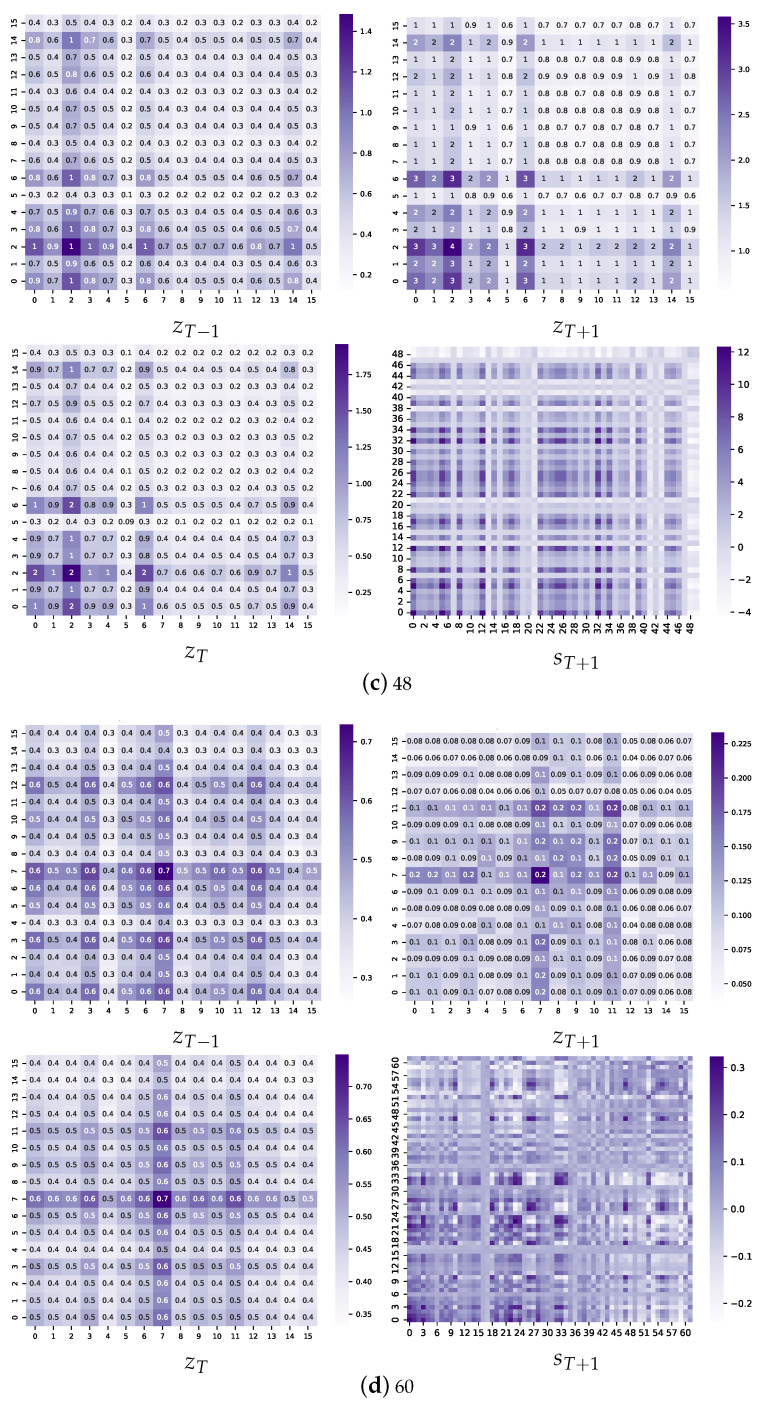
The covariance matrices for all the states of the prediction processes for the four tasks. Taking (**a**), for example, $z_{T-1}$, $z_{T}$, and $z_{T+1}$ are the adaptive covariance matrices for each period, and the heat map of prediction series is $s_{T+1}$.

SCACD outperforms other deep models in terms of prediction efficiency and interpretability, and it investigates the inference behind the adaptive covariance matrix and causal convolution. Taking the ILI data set as an example, Figure 5 shows the covariance matrices of *z* and *s* at each scale. First, the heat of the sub-diagonal region of each matrix is darker, indicating the topological relationship between the dimensions of similar latent variables and the higher correlation between variables with a similar relationship.

**Figure A2:**
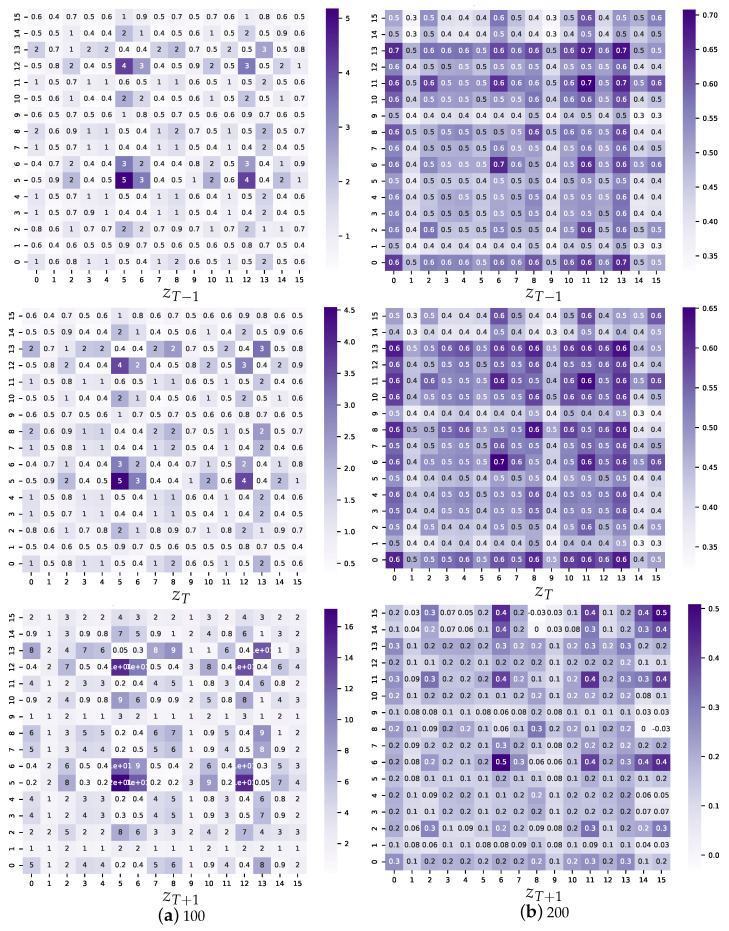

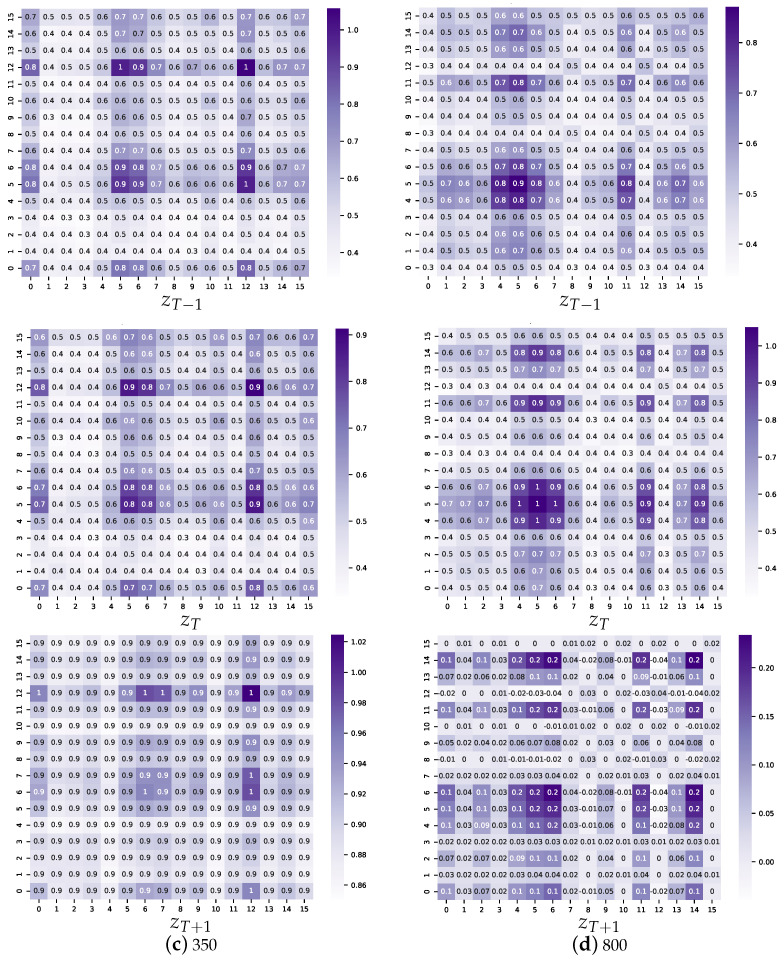
The covariance matrices for all the states for the ETT.

In addition, there are highly correlated elements in the off-diagonal region. For example, the correlation coefficient between dimension 10 and dimension 12 in $z_{T-1}$, Figure 5a, in which a total of 106 values are displayed, is 0.9, ranking second. The necessity of estimating covariance matrices for $z_{T-1}$ and $z_{T}$ is verified. Meanwhile, the rank changes smoothly. Consider again the 10–12 pairs mentioned above; the ranks in $z_{T-1}$, $z_{T}$, and $z_{T+1}$ are 2, 1, and 2, respectively. The index conforms to the concept drift principle, verifying the rationality and effectiveness of the state causal convolutional network. For larger scale time series, taking ETT as an example, the heat maps are shown in Figure A2.

The covariance matrix of the observation series is shown on the far right in Figure 5, and it has similar characteristics to the covariance matrix of the latent variable. The high correlation in off-diagonal regions also demonstrates the necessity of the covariance matrix-based sampling strategy.

### 4.6. Analysis for the Dimension of *z*

SCACD obtained excellent performance for long-term forecasting. The latent variable is essential for inferring the distribution of the observed sequence, so we conducted experiments to investigate the effect of the dimension of *z* on prediction precision. The dimensions of *z* are set as 8, 16, 24, and 32, and SCACD was implemented, respectively. The corresponding results in Table 3 show that the efficiency of the model with 16-dimensional *z* was better overall compared to the other settings. Generally, the prediction results for all four settings are good, indicating a fair and robust model.

### 4.7. Analysis of Efficiency

In comparison to the other deep models, SCACD obtains outstanding performance based on a simple construction. This experiment illustrates the proposed model’s indicators in the implementing processes. Table 4 shows the number of model parameters and the time required for the forward and backward processes under the same conditions.

**Figure 6:**
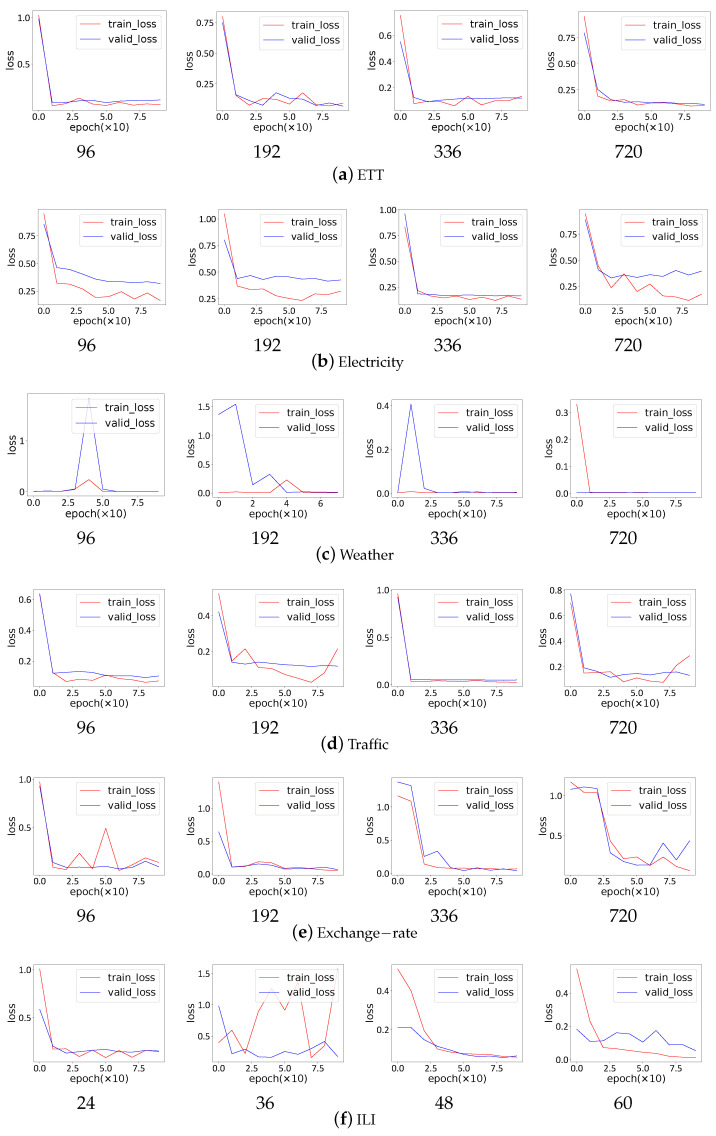
The convergence process of SCACD. The abscissa represents the number of epochs, and the ordinate represents the loss.

It is evident that SCACD has the fewest parameters and takes the least time. Furthermore, SCACD converges swiftly in all the prediction tasks shown in Figure 6, which demonstrates the model’s stability and robustness.

## 5. Conclusions

This work proposes a time-series prediction model (SCACD) based on state convolution and adaptive covariance matrix decomposition to handle the challenges of long-term prediction of large-scale time series. SCACD is a large-scale generation strategy to solve the cumulative error problem. First, MLPs are utilized to adaptively encode the hidden states. Secondly, a causal convolution is implemented to predict the distribution of future variables, and finally, a decomposition-based sampling is executed to complete the forecasting. SCACD infers the generational law of large-scale time series through two steps, as opposed to existing methods that rely on calculating the attention of the observation sequence. As it turned out, SCACD has significant advantages over baseline models, particularly in weak cycle datasets such as Exchange and ILI, where SCACD outperforms the current SOTA model by more than 500%. Furthermore, the extensive experiments demonstrate that the proposed model is highly interpretable and performs well.

Possible directions for extending this work include finding algorithms for extracting subsequences based on concept drift and multi-level causal convolution strategies. In this work, we used a single-level causal convolution network because of the fixed length of the subsequence and the small number of subsequences. Foreseeably, as the scale of the time series increases further, one could construct personalized multi-scale prediction models by combining adaptive subsequences, causal convolution, and dilated convolution [28]. 

## Figures and Tables

**Table 1:** The details of the datasets.

	Strongly Periodic Datasets
**ETT**	ETT is a key time-series indicator for long-term power deployment, which contains electricity data recorded every 15 min for two different counties in China from July 2016 to July 2018.
**Electricity**	This dataset contains the hourly electricity consumption of 321 customers from 2012 to 2014. It records electricity usage in kilowatt-hours and is timestamped every 15 min.
**Weather**	In order to verify the effect of the algorithm, we selected weather data containing 21 meteorological indicators (such as air temperature and humidity) in 2020 from the public dataset, and its time series sampling frequency was 10 min.
	**Weekly periodic datasets**
**Exchange**	Exchange records the daily exchange rates of eight different countries as a type of classical time series, with frequencies being daily, and the dataset covers exchange rate data from 1990 to 2016.
**Traffic**	Traffic is a collection of hourly data covering the highway system in all major urban areas of California, recording road occupancy rates measured by different sensors at different times of the day.
**ILI**	To verify the robustness of the algorithm on multiple time series datasets, we selected this dataset as the final part, which includes weekly Influenza-Like Illness (ILI) patient data recorded from 2002 to 2021, with the time series frequency being the weekly frequency data, which describes the ratio of ILI patients to the total number of patients.

**Table 2:** The best results are shown in bold. Compared with the current state-of-the-art FEDformer, the experimental effect of SCACD is improved by 10–100% in blue font, 100–500% in brown font, and greater than 500% in red font. The results for TCN are from Autoformer [22].

Models		SCACD		SCACD-nc		FEDformer		ARIMA		LSTM		TCN		DeepAR		GM11	
	**Len**	**MSE**	**MAE**	**MSE**	**MAE**	**MSE**	**MAE**	**MSE**	**MAE**	**MSE**	**MAE**	**MSE**	**MAE**	**MSE**	**MAE**	**MSE**	**MAE**
ETT	96	0.066	0.186	0.063	0.184	**0.035**	**0.146**	0.242	0.367	4.865	2.129	3.041	1.330	1.485	1.052	0.041	0.150
	192	0.075	0.215	0.082	0.225	**0.066**	**0.202**	0.147	0.284	4.094	1.952	3.072	1.339	3.871	1.772	0.886	0.243
	336	0.100	0.237	0.131	0.288	**0.088**	**0.234**	0.200	0.312	3.344	1.713	3.105	1.348	1.045	0.783	0.370	0.286
	720	**0.073**	**0.211**	0.139	0.280	0.106	0.252	0.138	0.249	3.413	1.676	3.135	1.354	0.998	0.825	0.096	0.236
Electricity	96	**0.213**	**0.340**	0.252	0.359	0.251	0.367	0.922	0.766	1.034	0.794	0.985	0.813	0.573	0.615	0.594	0.624
	192	0.378	0.463	0.380	0.463	**0.056**	**0.184**	1.047	0.801	0.723	0.668	0.996	0.821	0.502	0.576	3.375	0.806
	336	0.160	0.289	0.224	0.341	**0.082**	**0.225**	0.991	0.749	1.428	0.890	1.000	0.824	0.582	0.619	0.967	0.369
	720	**0.208**	**0.302**	0.566	0.545	0.403	0.473	3.298	0.990	0.559	0.622	1.438	0.784	0.671	0.675	1.166	0.636
Exchange	96	0.072	0.202	0.073	0.203	0.170	0.319	**0.059**	0.180	0.247	0.432	3.004	1.432	1.368	1.095	0.143	**0.129**
	192	**0.054**	0.191	0.054	0.182	0.311	0.431	0.259	0.370	0.412	0.514	3.048	1.444	0.275	0.431	0.087	**0.166**
	336	0.071	0.213	**0.066**	**0.204**	0.599	0.592	0.668	0.502	0.445	0.524	3.113	1.459	0.713	0.732	0.264	0.304
	720	**0.187**	**0.329**	1.331	0.980	1.432	0.924	1.536	0.934	0.945	0.826	3.150	1.458	0.282	0.424	0.839	0.550
Weather	96	**0.055**	**0.166**	0.081	0.204	0.211	0.323	0.500	0.579	0.960	0.855	1.438	0.784	0.973	0.837	1.029	0.880
	192	**0.118**	**0.232**	0.131	0.247	0.223	0.328	0.747	0.727	0.936	0.824	1.463	0.794	1.109	0.869	0.943	0.848
	336	**0.057**	0.171	0.059	**0.127**	0.220	0.326	0.734	0.712	0.964	0.839	1.479	0.799	0.834	0.807	0.925	0.839
	720	**0.102**	**0.230**	0.148	0.290	0.233	0.333	0.859	0.801	0.908	0.777	1.499	0.804	0.823	0.791	0.924	0.844
Traffic	96	**0.002**	**0.030**	0.005	0.057	0.005	0.056	0.005	0.052	0.040	0.153	0.615	0.589	0.125	0.282	0.414	0.062
	192	**0.001**	**0.022**	0.008	0.068	0.005	0.057	0.006	0.050	0.083	0.248	0.629	0.600	0.096	0.247	0.791	0.089
	336	0.002	0.029	**0.001**	**0.026**	0.005	0.054	1.666	0.095	0.179	0.399	0.639	0.608	0.124	0.280	0.798	0.094
	720	**0.002**	**0.035**	0.005	0.052	0.004	0.046	1.744	0.059	0.077	0.258	0.639	0.610	0.321	0.451	0.560	0.072
ILI	24	**0.111**	**0.284**	0.158	0.334	0.701	0.629	0.193	0.341	1.005	0.842	6.624	1.830	0.893	0.750	0.765	0.481
	36	**0.181**	0.356	0.187	**0.343**	0.581	0.613	0.258	0.399	1.098	0.852	6.858	1.879	0.948	0.763	0.279	0.371
	48	0.062	0.196	**0.054**	**0.168**	0.713	0.699	0.343	0.427	0.929	0.729	6.968	1.892	1.045	0.803	0.296	0.361
	60	**0.065**	**0.193**	0.197	0.327	0.840	0.768	0.229	0.379	0.789	0.665	7.127	1.918	1.094	0.818	0.851	0.443
																	
			10–100%				100–500%				>=500%						

**Table 3:** The dimensions of the latent variable *z* in SCACD are taken as 8, 16, 24, and 32. The other hyperparameters are as in Section 4.1.

The Dim of Latent		SCACD-8		SCACD-16		SCACD-24		SCACD-32	
	**Len**	**MSE**	**MAE**	**MSE**	**MAE**	**MSE**	**MAE**	**MSE**	**MAE**
ETT	96	0.064	0.185	0.066	0.186	0.088	0.216	**0.063**	**0.180**
	192	0.140	0.296	**0.075**	**0.215**	0.126	0.280	0.116	0.262
	336	**0.099**	0.250	0.100	**0.237**	0.148	0.290	0.138	0.288
	720	0.189	0.344	**0.073**	**0.211**	0.225	0.368	0.149	0.310
Electricity	96	0.291	0.403	**0.213**	**0.340**	0.317	0.410	0.252	0.366
	192	0.458	0.523	**0.378**	0.463	0.387	0.473	0.379	**0.460**
	336	0.212	0.333	**0.160**	**0.289**	0.261	0.365	0.255	0.380
	720	0.375	0.466	**0.208**	**0.302**	0.504	0.499	0.483	0.527
Exchange	96	0.078	0.206	0.072	0.202	**0.044**	**0.165**	0.057	0.197
	192	0.138	0.297	**0.054**	**0.191**	0.261	0.369	0.063	0.195
	336	0.405	0.501	**0.071**	**0.213**	0.128	0.280	0.564	0.581
	720	0.757	0.624	0.908	0.780	0.796	0.844	**0.509**	**0.597**
Traffic	96	0.095	0.203	**0.055**	**0.166**	0.087	0.197	0.087	0.199
	192	0.100	0.217	0.118	0.232	**0.077**	**0.201**	0.165	0.275
	336	**0.035**	**0.117**	0.057	0.171	0.044	0.130	0.047	0.133
	720	0.132	0.262	**0.102**	**0.230**	0.168	0.281	0.144	0.267
Weather	96	**0.001**	**0.023**	0.002	0.030	0.006	0.056	0.018	0.075
	192	0.004	0.050	**0.001**	**0.022**	0.001	0.023	0.004	0.050
	336	0.005	0.071	**0.002**	**0.029**	0.003	0.045	0.003	0.044
	720	0.005	0.039	**0.002**	**0.035**	0.215	0.070	0.036	0.144
ILI	24	0.094	0.260	0.111	0.284	0.129	0.294	**0.094**	**0.242**
	36	**0.170**	**0.336**	0.181	0.356	0.187	0.358	0.186	0.346
	48	0.135	0.304	**0.062**	**0.196**	0.167	0.344	0.117	0.276
	60	0.088	0.231	0.065	0.193	0.117	0.261	**0.029**	**0.132**

**Table 4:** The values correspond to the prediction scales. The hyperparameters are set as in Section 4.1. P: the number of the parameters; B: time (s/batch) for the backward; F: time (s/batch) for the forward.

	96			192			336			720		
	P	B	F	P	B	F	P	B	F	P	B	F
SCACD	$4.85\times 10^{4}$	$2.74\times 10^{-2}$	$1.18\times 10^{-2}$	$1.74\times 10^{5}$	$2.68\times 10^{-2}$	$1.17\times 10^{-2}$	$5.13\times 10^{5}$	$3.03\times 10^{-2}$	$1.15\times 10^{-2}$	$2.61\times 10^{6}$	$4.10\times 10^{-2}$	$1.50\times 10^{-2}$
Autoformer	$1.1\times 10^{7}$	$6.63\times 10^{-2}$	$6.59\times 10^{-2}$	$1.05\times 10^{7}$	$1.04\times 10^{-1}$	$1.23\times 10^{-1}$	$1.05\times 10^{7}$	$1.91\times 10^{-1}$	$2.36\times 10^{-1}$	$1.05\times 10^{7}$	$1.07\times 10^{-1}$	$1.17\times 10^{-1}$
FEDformer	$1.63\times 10^{7}$	$2.54\times 10^{-1}$	$5.50\times 10^{-2}$	$1.63\times 10^{7}$	$2.51\times 10^{-1}$	$5.83\times 10^{-2}$	$1.68\times 10^{7}$	$2.87\times 10^{-1}$	$6.73\times 10^{-2}$	$1.63\times 10^{7}$	$2.93\times 10^{-1}$	$6.69\times 10^{-2}$

## Data Availability

The datasets’ sources are fully annotated in the paper.

## Appendix A

### Appendix A.1. Prediction Curves

The prediction results prove that SCACD has mastered the time-series generation law, which considerably decreases the accumulation of long-term prediction errors in large-scale time series. The prediction curves are shown in Figure A1.

### Appendix A.2. Heatmaps for ETT

Due to the large-scale characteristic, the covariance matrix heat map of *s* has poor resolution, so only the covariance matrices of the latent variables are shown in Figure A2.
